# Staphylococcus caprae Infections in Neonatal Intensive Care Units: A Report of Two Cases

**DOI:** 10.7759/cureus.74124

**Published:** 2024-11-20

**Authors:** Mafalda J Pereira, Andreia Fernandes, Íris Oliveira, Cláudia Calado, Luísa Gaspar

**Affiliations:** 1 Pediatrics, Unidade Local de Saúde do Algarve, Faro, PRT

**Keywords:** late-onset sepsis, neonatal intensive care unit (nicu), neonatal sepsis, preterm neonate, staphylococcus caprae

## Abstract

*Staphylococcus caprae *(*S. caprae*) is a coagulase-negative staphylococci. This group of bacteria is typically part of the skin flora but can become pathogenic in susceptible hosts, such as preterm infants with prolonged stays in neonatal intensive care units. Two preterm newborns with late-onset sepsis caused by *S. caprae*, one of whom also developed meningitis. Antibiotics were initiated, and both infants had favorable outcomes. While *S. caprae* is a well-documented agent of infection in adults, there are only rare reports of infection in neonates, particularly in preterms. Differentiating between contamination and infection is crucial for the appropriate initiation of antibiotics.

## Introduction

Coagulase-negative staphylococci (CoNS) are part of the bacterial population in the skin, and represent one of the major causes of bacteremia and other infections in intensive care units, including neonatal units (NICU) [1-4].

*Staphylococcus caprae* (*S. caprae*) is a catalase-positive CoNS; an organism usually associated with animals. Most documented cases of infection are hospital-acquired bone and joint infections in adults, although there have been rare case reports of infection in newborns [1,5,6].

Despite improvements in neonatal care and the adoption of quality enhancement strategies, late-onset sepsis (LOS) continues to be a significant threat in the NICU. LOS primarily impacts most premature infants and is linked to both high mortality rates and serious long-term health complications in survivors. Identifying and diagnosing LOS remains difficult, as it often presents with a range of nonspecific clinical symptoms, and common laboratory markers are not always reliable in distinguishing between infected and uninfected infants [4,6].

In this article, we report two cases of premature newborns admitted to the NICU, with infections caused by *S. caprae*.

## Case presentation

Case 1

A male newborn delivered at 33+1 weeks of gestational age, with a birth weight of 2215 grams (+ 0.51 SD). The mother was Portuguese, unemployed, and healthy. The pregnancy was complicated by gestational diabetes and preeclampsia, managed with aspirin. Routine serological tests (hepatitis B virus, human immunodeficiency virus, syphilis, toxoplasmosis, rubella, and cytomegalovirus) were negative, and ultrasounds were normal (Table 1). The baby was delivered by emergency cesarean section due to premature labor and placenta previa. He was born in good condition, with an Apgar score of 7 in the first minute, 8 after five minutes and 9 by the 10th-minute mark.

**Table 1:** Tests performed in each case N.A.: not applicable; R.R.: reference range; CSF: cerebrospinal fluid

Study performed	Case 1	Case 2	R.R.
During pregnancy (first, second and third trimesters)			
Hepatitis B surface antigen (HBsAg) and hepatitis B surface antibody (anti-HBs)	Negative	Negative	N.A.
Human immunodeficiency virus (HIV) type 1 and 2 antigen (Ag)/antibody (Ab) combination screen	Negative	Negative	N.A.
Rapid plasma reagin (RPR) test	Negative	Negative	N.A.
Toxoplasma serology (IgG, IgM)	IgG negative, IgM negative	IgG negative, IgM negative	N.A.
Rubella serology (IgG, IgM)	IgG positive, IgM negative	IgG positive, IgM negative	N.A.
Cytomegalovirus serology (IgG, IgM)	IgG negative, IgM negative	IgG positive, IgM negative	N.A.
Prenatal ultrasound	No abnormalities	No abnormalities	N.A.
After birth (first day of life)			
Leukocyte count	17,560	15,480	9,000-30,000 cells/μL
C-reactive protein	Negative	Negative	<1 mg/L
Blood culture (two in each case)	Negative	Negative	N.A.
After birth	8^th^ day of life	5^th^ day of life	
Leukocyte count	37,730	25,000	9,000-30,000 cells/μL
C-reactive protein	45.0 mg/L	33 mg/L	<1 mg/L
Procalcitonin	18.2 ng/mL	N.A.	<0.5 ng/mL
Blood culture (two in each case)	Both are positive for *Staphylococcus caprae*	Both positive for *S. caprae*	N.A.
CSF cytology	Negative	N.A.	N.A.
CSF culture	Positive for *S. caprae*	N.A.	N.A.

He was admitted to the NICU and continuous positive airway pressure (CPAP) was initiated. Due to worsening respiratory distress and increasing oxygen requirements, he initially received less invasive surfactant administration but later required tracheal intubation.

On the first day of life, ampicillin and gentamicin were started. Both blood cultures and inflammatory markers were negative (Table 1), and antibiotics were discontinued after five days.

On day 8 of life, he developed a fever (maximum temperature of 38.7ºC). Both C-reactive protein (CRP) and procalcitonin levels were elevated (Table 1). Cerebrospinal fluid (CSF) analysis was unremarkable. The patient was started on vancomycin and gentamicin. After four days, two blood cultures and one CSF culture returned positive for *S. caprae*. Antibiotic susceptibility testing could not be performed due to the lack of an appropriate panel for this bacterium. As the infant remained clinically unwell, antibiotics were changed to vancomycin and cefotaxime. He completed a 10-day course of the final antibiotic regimen, after which he was clinically well, with normalized inflammatory markers. A central catheter was not necessary during the NICU admission. He was discharged at 36+3 weeks of post-menstrual age (24 days of life) and is reported to be clinically well at regular outpatient follow-ups.

Case 2

A female newborn delivered at 33 weeks of gestational age, with a birth weight of 1537 grams (-0.93 SD). The mother was Indian, unemployed, and healthy. The pregnancy was unremarkable, with normal ultrasounds, and negative serologies (Table 1). The baby was delivered by emergency cesarean section due to premature labor and breech presentation. The Apgar score was 7 in the first minute, 8 after five minutes and 9 by the 10th-minute mark. She was started on CPAP for respiratory distress. Her initial inflammatory parameters and blood cultures were negative (Table 1), so she did not receive antibiotics.

On day 5 of life, she became lethargic, with blood tests showing an elevated CRP (Table 1), and was started on ampicillin and gentamicin. A peripherally inserted central catheter was placed on the sixth day of life (after the infection was noticed and antibiotics were started), and was removed after 21 days. She improved clinically over the next few days. After six days, two blood cultures returned positive for *S. caprae*. Antibiotic susceptibility tests were not possible to perform in the microbiology laboratory of the hospital. Due to her clinical improvement, antibiotic therapy was not changed and she completed 12 days of the antibiotic course. She was discharged with 36+5 weeks of postmenstrual age (27 days of life) and is reported to be clinically well at regular outpatient follow-ups.

These two cases occurred approximately two months apart in the same NICU. There were no other positive cultures for *S. caprae* in the following eight months.

## Discussion

We report two preterm infants with LOS due to *S. caprae*, one of whom also had meningitis. These cases occurred in the same NICU, approximately two months apart. Although a link between them was not established and no other cases emerged, we cannot exclude a connection.

LOS is primarily linked to nosocomial infections. Exposure to pathogens can occur through the contamination or colonization of medical devices, contact with healthcare workers, or contact with other environmental sources and surfaces. Premature birth and severe illness are significant risk factors for LOS, as they often require the use of central catheters, mechanical ventilation, prolonged parenteral nutrition, and sometimes surgery. Other contributing factors include maternal and perinatal conditions such as preeclampsia, chorioamnionitis, intrauterine growth restriction, as well as the duration of hospitalization and existing comorbidities. Most premature infants bear the highest risk of infection. LOS-associated mortality rates vary depending on gestational age and the type of pathogen involved, reaching as high as 35% in the most vulnerable, extremely premature infants. LOS is often caused by gram-positive organisms. Over 50% of gram-positive bacteremia in preterm infants is due to CoNS (in which *S. caprae* is included), which are usually considered skin commensals in term infants. However, in preterm neonates, CoNS can be considered true pathogens causing infections that are clinically significant, but always posing the question of true bacteremia versus contamination [4,6-8]. Clinical presentation and several positive cultures are essential for the diagnosis.

In our report, multiple cultures (two blood cultures in each case and CSF culture in the first case) were positive for *S. caprae* in clinically unwell infants with elevated inflammatory markers, indicating likely true infection rather than contamination. Beta-lactam antibiotics are commonly utilized to cover gram-positive bacteria, and most *S. caprae* are methicillin-sensitive. However, high rates of resistant bacteria in NICU have been described, so vancomycin may be considered for initial gram-positive coverage. Factors influencing the choice of empiric antibiotics include the presence of central catheters, known colonization with methicillin-resistant *S. aureus* (MRSA), and local resistance patterns. Vancomycin use in NICU is widespread and is among the most commonly used antibiotics [3-6]. In our unit, since MRSA infection is common, vancomycin is considered empirically in neonates with previous antibiotic use. In both our reports, antibiotic sensitivity testing was not performed, so antibiotic adjustment was based on clinical response. In the first case, the initial clinical response was slow, prompting the change to vancomycin and cefotaxime; in the second case, the clinical response was favorable, so the infant continued on ampicillin and gentamicin. Both cases progressed well.

In adults, risk factors for *S. caprae* infection include diseases such as diabetes mellitus, chronic renal failure, immunodeficiencies, open fractures, and malignancies; biological factors such as the male gender; iatrogenic factors such as implanted devices, recent antibiotic use, chemotherapy and radiation therapy, and immunosuppression associated with systemic or local corticosteroids; and social factors like sheep or goat proximity. In the pediatric population, specifically in neonates, no specific risk factors for this pathogen are known [2,3,9,10].

Traditional methods of phenotypic identification frequently fail to accurately recognize the extent of *S. caprae* infections. These are infrequently reported possibly due to the absence of fast and sensitive instruments. Many laboratories do not routinely identify CoNS species, so the real incidence linked to *S. caprae* is unknown, along with the ability to predict antibiotic susceptibility [1,5]. The virulence of these bacteria is connected to their potential to create biofilm and colonize biological tissues and materials [2,8,11].

Neonatal case reports of *S. caprae* infection are rare [1]. In Ross TL et al. (2005), *S. caprae *was detected in specimens from six infants in a NICU, identified in an MRSA detection study. Additionally, they reported six cases of bacteriemia and two of venous catheter and CSF shunt colonization amounting to eight potential infections [1]. In two other case reports, single cases of *S. caprae* sepsis occurred in neonates with congenital heart disease [12,13]. In all these descriptions, no fatal cases were reported [1,12,13].

## Conclusions

Although *S. caprae* infection is rare, premature babies with inherent immunosuppression are a group at risk for this infection. In our unit, these two babies were the first cases of known infection by this organism, with added difficulty in antibiotic management due to unknown susceptibility patterns. The cases described in the literature are scarce, and without antibiotic susceptibility tests, treatment must be based on CoNS empirical antibiotic recommendations.
